# The impact of vitamin D changes during pregnancy on the development of maternal adverse events: a random forest analysis

**DOI:** 10.1186/s12884-024-06294-5

**Published:** 2024-02-10

**Authors:** Nasrin Borumandnia, Maryam Rostami, Atefeh Talebi, Hamid Alavimajd

**Affiliations:** 1 Urology and Nephrology Research Centre, Shahid Beheshti University of Medical Sciences, Tehran, Iran; 2 Department of Epidemiology and Biostatistics, Tehran Medical Sciences Branch, Islamic Azad University, Tehran, Iran; 3 Department of Social Medicine, Faculty of Medicine, Ahvaz Jundishapur University of Medical Sciences, Ahvaz, Iran; 4 British Heart Foundation Cardiovascular Research Centre, University of Glasgow, Glasgow, UK; 5 Department of Biostatistics, School of Allied Medical Sciences, Shahid Beheshti University of Medical Sciences, Tehran, Iran

**Keywords:** Maternal adverse events, Vitamin D, Random forest

## Abstract

**Background:**

Maternal vitamin D deficiency during pregnancy has been associated with various maternal adverse events (MAE). However, the evidence regarding the effect of vitamin D supplementation on these outcomes is still inconclusive.

**Methods:**

This secondary analysis utilized a case–control design. 403 samples with MAE and 403 samples without any outcomes were selected from the Khuzestan Vitamin D Deficiency Screening Program in Pregnancy study. Random forest (RF) analysis was used to evaluate the effect of maternal vitamin D changes during pregnancy on MAE.

**Results:**

The results showed that women who remained deficient (35.2%) or who worsened from sufficient to deficient (30.0%) had more MAE than women who improved (16.4%) or stayed sufficient (11.8%). The RF model had an AUC of 0.74, sensitivity of 72.6%, and specificity of 69%, which indicate a moderate to high performance for predicting MAE. The ranked variables revealed that systolic blood pressure is the most important variable for MAE, followed by diastolic blood pressure and vitamin D changes during pregnancy.

**Conclusion:**

This study provides evidence that maternal vitamin D changes during pregnancy have a significant impact on MAE. Our findings suggest that monitoring and treatment of vitamin D deficiency during pregnancy may be a potential preventive strategy for reducing the risk of MAE. The presented RF model had a moderate to high performance for predicting MAE.

## Introduction

Maternal vitamin D deficiency during pregnancy has been associated with various adverse maternal–fetal outcomes [1, 2]. Vitamin D, a steroid hormone, is responsible for the regulation of calcium and phosphate homeostasis. Additionally, it assumes a pivotal function in a multitude of physiological processes, encompassing calcium and bone metabolism, immune function, and cell proliferation [3–5]. Vitamin D insufficiency has been correlated with an elevated susceptibility to gestational diabetes, premature delivery, and postpartum hemorrhage [6]. The exact mechanisms underlying these associations are not fully understood, but some studies suggest that vitamin D has been linked to characteristics that counteract inflammation and oxidation, potentially mitigating the occurrence of oxidative stress and inflammation, both of which are established precursors for undesirable outcomes experienced by mothers [2, 7]. Moreover, vitamin D might have a role in the regulation of immune function and the prevention of autoimmune disorders while a woman is pregnant [8]. However, excessive vitamin D intake has also been associated with adverse outcomes, such as hypercalcemia and nephrolithiasis. Further investigations are required to establish a cause-and-effect association and identify the most effective means of supplementation [9].

Supplementation of Vitamin D throughout the pregnancy has demonstrated positive effects on the health outcomes of both the mother and the newborn. It notably augments the concentrations of 25-hydroxyvitamin D (25(OH)D) in both the maternal and infant bodies, potentially contributing to the occurrence of maternal insulin resistance as well as the growth of the fetus [10]. Although vitamin D insufficiency has been associated with maternal complications, the evidence regarding the effect of vitamin D supplementation on these outcomes is still inconclusive [11, 12]. Vitamin D supplementation during pregnancy has shown mixed and controversial results in improving these outcomes [13, 14]. Some studies suggest that supplementation may improve birth length, operative delivery and preeclampsia [11, 12] and monitoring and treatment have been recommended during pregnancy [15]. While others have not found significant associations with these outcomes or have mentioned limited evidence on the effects of vitamin D supplementation [11, 12, 16, 17]. Therefore, the actual benefit of vitamin D supplementation in reducing the incidence of adverse outcomes has been remained unclear, and more research is needed. In this study, we conducted a secondary analysis on a subset of data from Khuzestan Vitamin D Deficiency Screening Program in Pregnancy (KVDSPP) study [18]. We used random forest (RF) approach to evaluate the effect of maternal vitamin D changes during pregnancy on MAE. To our knowledge, no specific studies using this approach were identified in our literature review. Our study aims to fill this gap by examining the influence of vitamin D status (VDS) during pregnancy on MAE using RF approach.

## Method

This secondary analysis utilized a case–control design. 403 samples with MAE and 403 samples without any outcomes were selected from the KVDSPP study. KVDSPP study was a two-phase study, a population-based cross-sectional study and a randomized controlled trial. The cross-sectional study assessed the prevalence of vitamin D deficiency in the first trimester of pregnancy, while the randomized controlled trial evaluated the effects of type, dose and duration of vitamin D supplementation on vitamin D deficiency and also on maternal and neonatal outcomes. The details of the study design and methods have been published elsewhere [18, 19]. The presented study aims to investigate whether vitamin D changes during pregnancy affect maternal complications.

The inclusion and exclusion criteria of the original KVDSPP study to select the participants were used for our secondary analysis. The inclusion criteria were: pregnant women aged 18–40 years, singleton pregnancy, gestational age of < 14 weeks, not consuming multivitamins containing > 400 IU/d of D3, and no previous history of chronic diseases (e.g., diabetes, hypertension, renal dysfunction, liver diseases, thyroid diseases, and complicated medical or obstetrical history), and residence in Khuzestan province. Participants were excluded if they consumed multivitamins containing more than 400 international units (IU) per day of vitamin D3; used anticonvulsants; and had a history of chronic diseases like diabetes, hypertension, renal dysfunction, liver diseases, and complicated medical or obstetrical history; and unwillingness to participate or follow the study protocol.

We excluded samples who had missing data. Therefore, this secondary analysis included 806 samples, of which 403 had MAE and 403 had no any outcomes. All these samples had complete information and no missing data.

First the women’s vitamin D levels were categorized into severely deficient (< 10 ng/mL), moderately deficient (10 to 20 ng/mL), and sufficient (> 20 ng/mL). Based on their vitamin D levels at the beginning and end of pregnancy, the samples were further divided into four subgroups: ‘Remained deficient’, ‘Improved from deficient to sufficient’, ‘Remained sufficient’, and ‘Worsened from sufficient to deficient’. The Table 1 shows the definition of each group used in the study. Women who had sufficient vitamin D levels at both the beginning and end of pregnancy were assigned to the ‘Remained sufficient’ subgroup and were colored dark green in the table. Women who had severely or moderately deficient vitamin D levels at both time points were assigned to the ‘Remained deficient’ subgroup and were colored yellow in the table. ‘Worsened’ group include those samples had vitamin D levels moderately deficient at the start of pregnancy, but severely deficient at the end of pregnancy; and also, those samples had vitamin D levels sufficient at the start of pregnancy, but moderately deficient or severely deficient at the end of pregnancy. They were colored light red in the table. Finally, women who had severely deficient vitamin D levels at the beginning of pregnancy, but improved to moderately deficient or sufficient levels by the end of pregnancy, and also those samples had vitamin D levels Moderately deficient at the start of pregnancy, but sufficient at the end of pregnancy were assigned to the “Improved” subgroup and were colored light green in the table.

**Table 1:**
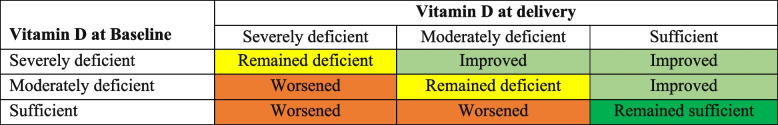
Grouping of mothers by vitamin D status at baseline and delivery. Same color indicates same group

The main dependent variable in this study was the occurrence of MAE during pregnancy and was defined as having any of the following conditions: preeclampsia (systolic blood pressure ≥ 140 mmHg or diastolic blood pressure ≥ 90 mmHg and 24-h proteinuria ≥ 0.3 g, started at > 20 weeks), abortion, gestational diabetes mellitus (GDM) (glucose intolerance first detected during pregnancy using criteria of the International Association of the Diabetes and Pregnancy Study Groups), preterm delivery (birth at < 37 weeks), and premature rupture of membranes (PROM) (rupture or breaking of the amniotic sac that surrounds and protects the fetus in the womb before the onset of labor or before 37 weeks of gestation) [18].

The impact of VDS during pregnancy on the aforementioned outcomes was investigated, along with the inclusion of other predictors such as maternal age, parity, baby sex, the average of systolic blood pressure (SBP) and diastolic blood pressure (DBP) throughout the pregnancy, and the percentage of pregnancy weight gain (PWG).

### Statistical analysis

The data were described using descriptive statistics and histogram, trend line plot, bar charts, and error bars. The frequency and percentage for categorical variables and the mean and SD for continuous variables were calculated. RF approach was employed to examine how VDS and other predictors such as maternal age, parity, sex of the baby, blood pressure, and PWG percentage during pregnancy affect MAE. The RE model is a type of powerful and robust machine learning algorithm that is commonly used in predictive modeling and classification tasks. It utilizes a collection of decision trees to predict the outcome. In this study, the main outcomes were binary, making the RF model suitable for addressing the research questions, which were to evaluate the effect of maternal vitamin D changes during pregnancy on MAEs. To further assess the performance of the RF model, a sensitivity analysis was conducted by comparing it with the traditional statistical methods, such as logistic regression (not shown here). The RF model outperformed logistic regression in terms of model performance, indicating that it was a more appropriate method for predicting MAE in our study population. Given its the proven performance, flexibility, and ability to handle complex data structures, the RF model was employed. The RF model was evaluated using different metrics, such as AUC, CA, F1 score, precision, sensitivity, and specificity. 5-fold cross-validation was conducted to validate the model. The purpose of cross-validation is to evaluate the performance of the model on unseen data and to estimate its generalization ability. 5-fold cross-validation involves splitting the available dataset into multiple (5 here) equal subset, called fold, and iteratively training and testing the model on four folds and using the remaining fold for validation. Finally, the results from each validation step are averaged to produce a more robust estimate of the model’s performance. The variables were ranked according to how important they are in models that the RF build. The assessment of the importance of variables in RF models was executed by means of employing the mean decrease impurity (MDI) technique. This particular approach calculates the importance of a variable by examining the improvement in the split criterion at each node where the variable is chosen for splitting. The progress is accumulated across all the trees within the forest and subsequently averaged. We did not consider a threshold for the importance of variables, as no variable selection was carried out based on the importance scores. The significance of all variables was duly reported. Orange3 version 3.21.0 software were utilized for data analysis.

## Results

The study selected 806 samples, of which half exhibited MAE and the other half showed no outcomes. Out of the 403 MAE specimens, preeclampsia manifested in 204 samples, abortion in 34, GDM in 79, preterm delivery in 200, and PROM in 198 samples. The characteristics of both samples with MAE as well as their controls, were reported in Table 2.

**Table 2:** The characteristics of the study participants

Feature	Maternal adverse events	
	Yes (*n* = 403)	No (*n* = 403)
Mother Age		
< 20	35(54.7%)	29(45.3%)
20–30	163(51.9%)	151(48.1%)
> 30	205(47.9%)	223(52.1%)
Mother job		
Employed	285(50.1%)	284(49.9%)
Housewife	118(49.8%)	119(50.2%)
PWG percentage		
< = 10%	47(81.0%)	11(19.0%)
10–20%	342(51.1%)	327(48.9%)
20–30%	14(17.7%)	65(82.3%)
SBP means		
= < 117	97(27.2%)	260(72.8%)
> 117	306(68.2%)	143(31.8%)
DBP means		
= < 70	146(34.1%)	282(65.9%)
> 117	257(68.0%)	121(32.0%)
Parity		
0	133(48.5%)	141(51.5%)
> 0	270(50.8%)	262(49.2%)
Baby sex		
Boy	202(49.0%)	210(51.0%)
Girl	201(51.0%)	193(49.0%)

*SBP means* Means of systolic blood pressure during pregnancy, *DBP means* Means of diastolic blood pressure, *PWG* Pregnancy weight gain

**Fig. 1:**
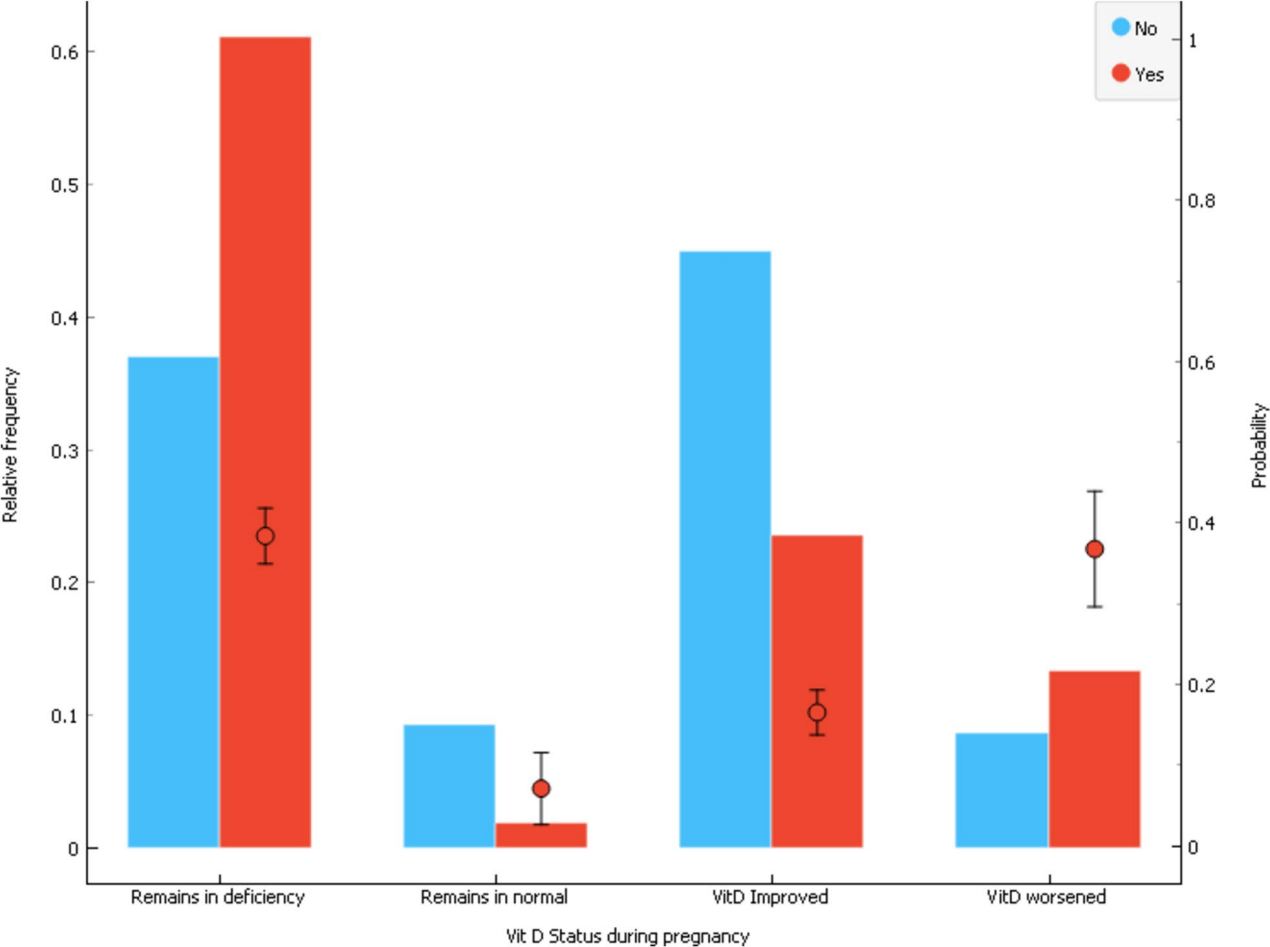
Distribution of vitamin D status during pregnancy by maternal adverse events. The bar charts show the relative frequency of vitamin D status among the women with presence and absence of adverse events, separately (left y axis, labeled relative frequency). The error bars show the 95% confidence intervals for the percentages of adverse event in each category of vitamin D status (right y axis, labeled probability)

The charts in Fig. 1 show the distribution of VDS among the pregnant women who participated in the study and show how the VDS of pregnant women during the pregnancy affects the occurrence of MAE. The x-axis shows the four categories of VDS. The bar charts show the relative frequency of VDS groups among the women with presence and absence of MAE, and it is corresponding to the left y axis, labeled relative frequency. The bar plots show that the majority of women with MAE belonged to the ‘remained deficient’ group (65%), and the fewest of them were in the ‘remained sufficient’ group (2.5%). The error bars show the 95% confidence intervals for the percentages of MAE in each category of VDS (right y axis, labeled probability). Considering the error bars, women who remained deficient (35.2%) or who worsened from sufficient to deficient (30.0%) had more MAE than women who improved (16.4%) or stayed sufficient (11.8%).

**Fig. 2:**
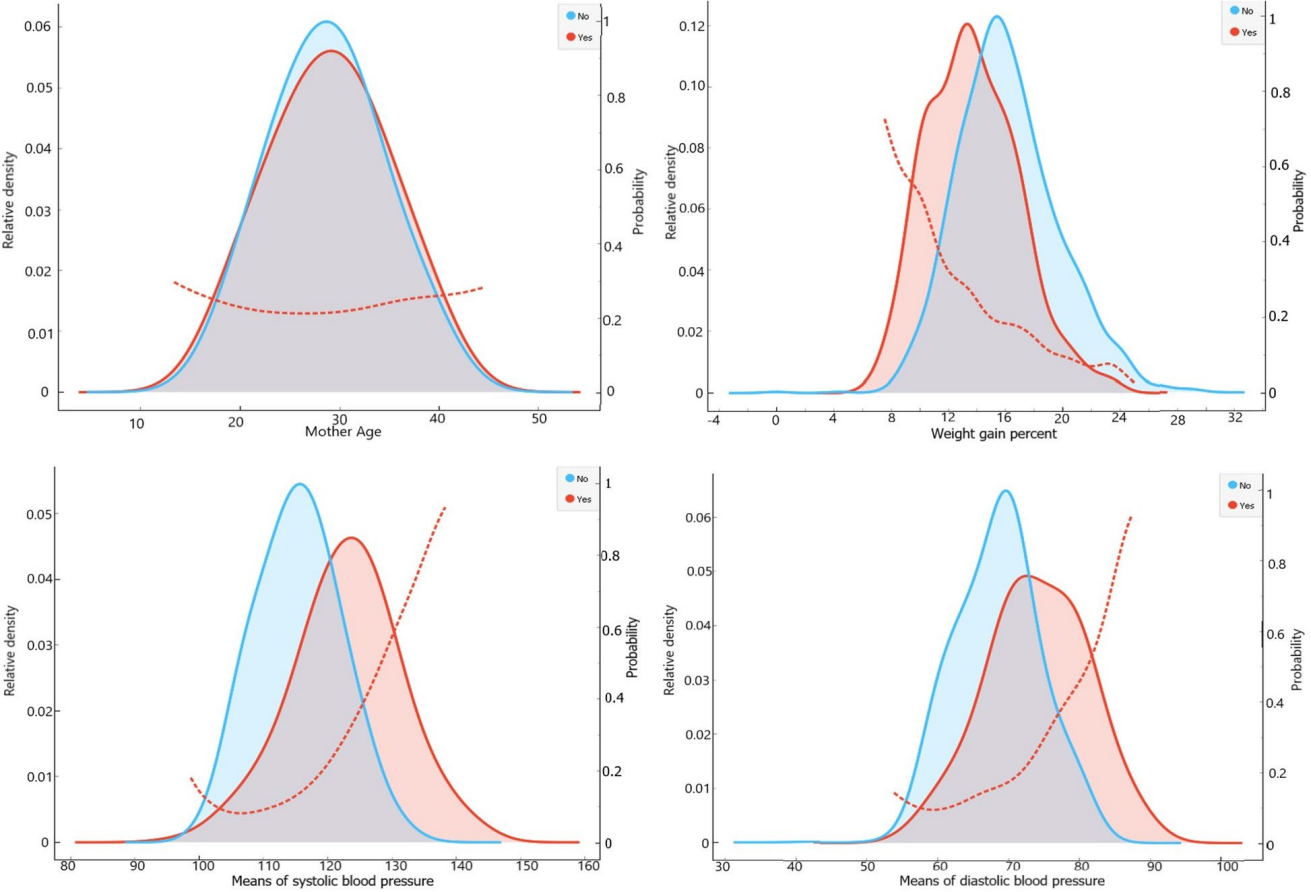
Distribution of mother age, pregnancy weight gain percent, means of systolic and diastolic blood pressure during pregnancy by maternal adverse event

Figure 2 shows the distribution of the mother’s age, PWG percentage, the mean of SBP and DBP during the pregnancy by MAE. Each plot has two curves, one in blue for women without MAE and one in red for women with MAE. The two curves represent the distribution of continuous variables separately for women with or without adverse events. For example, comparing the two curves of SBP shows that women with MAE have a higher mean of SBP than women without MAE, which means that the average value of the SBP in women with MAE is larger than the average value in women without MAE.

The dotted line compares the probability of MAE in different levels of the continuous variable. The graph indicates that the probability of MAE increases as the SBP and DBP increases. For example, at a mean SBP of 110 mmHg, the probability of MAE is about 0.1 and the probability increases to 0.2 when the mean SBP reaches 120 mmHg. Also, the graphs suggest that low PWG percentage is a risk factor for MAE, and the mother’s age does not affect the probability of MAE as much as the effect of other factors.

**Fig. 3:**
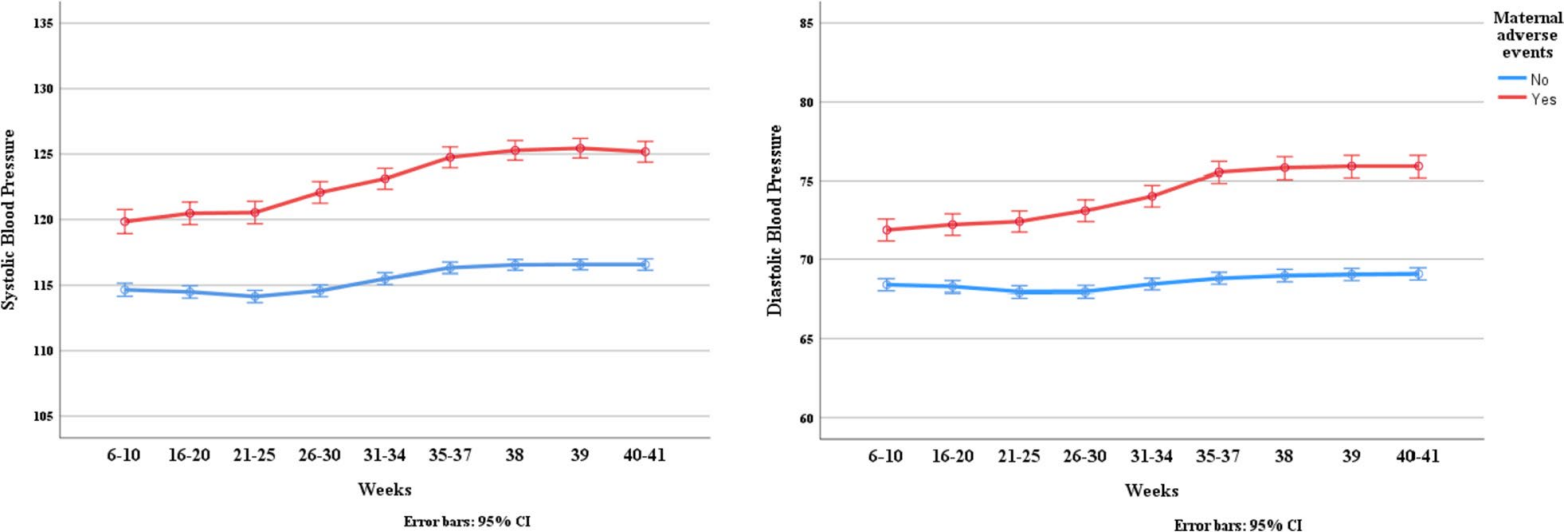
The trend of systolic and diastolic blood pressure means by maternal adverse events

Figure 3 illustrates the changes in SBP and DBP means throughout pregnancy by MAE status. The plot indicates that blood pressure increases as pregnancy advances in both groups of women. However, women who experienced an MAE had higher SBP and DBP than those who did not.

Then a RF model was used to examine how VDS and other factors affect MAE. Table 3 reported the performance metrics for each fold and the overall mean. The results show that the RF model achieved a high and consistent performance across the folds, with an average AUC of 0.74, sensitivity of 72.6%, specificity of 69%, precision of 0.68, CA of 0.69, and F1 of 0.70. The standard deviations of these metrics were also low and individual fold varied slightly, indicating a low variability in the model performance. These results suggest that the RF model has good predictive performance and generalization ability, and that it can accurately predict maternal adverse events based on clinical and demographic factors.

**Table 3:** Performance metrics across different folds and the overall mean

Fold	AUC	Sensitivity	Specificity	Precision	CA	F1
1	0.742	0.725	0.695	0.684	0.695	0.704
2	0.743	0.734	0.698	0.671	0.687	0.701
3	0.736	0.725	0.683	0.682	0.694	0.703
4	0.740	0.717	0.678	0.671	0.682	0.682
5	0.739	0.727	0.697	0.693	0.702	0.709
Overall mean	0.74	0.726	0.690	0.680	0.692	0.70
SD	0.003	0.006	0.009	0.009	0.008	0.01

Table 4 shows how different predictors affect the risk of MAE (having one or more events) and each event individually. The predictors were ranked based on their influence in the RF models. The higher the rank, the more important the predictor was. For the overall MAE, the table reveals that SBP was the most important predictor, followed by DBP and VDS during pregnancy. Notably, VDS also played a significant role in other outcomes. The main factors for preeclampsia were the average SBP and DBP, with VDS during pregnancy as the third predictor. The main factors contributing to abortion were the mother age, VDS during pregnancy and mother job. The main factors for GDM were the PWG percentage, mother age and VDS during pregnancy. The primary determinants for preterm delivery were the average SBP, VDS and the average DBP during pregnancy. The main factor for PROM was VDS, followed by the average SBP and DBP during pregnancy.

**Table 4:** Variable importance based on mean decrease impurity score for predictors of Maternal adverse events including preeclampsia, abortion, gestational diabetes mellitus, preterm delivery, or premature rupture of membranes

Rank	Maternal adverse events		Preeclampsia		Abortion		GDM		Preterm delivery		PROM	
	**Variable**	MDI	**Variable**	MDI	**Variable**	MDI	**Variable**	MDI	**Variable**	MDI	**Variable**	MDI
**1**	SBP	.206	SBP	.284	Maternal age	.164	PWG	.079	SBP	.117	VDS	.179
**2**	DBP	.114	DBP	.218	VDS	.151	Maternal age	.074	VDS	.115	SBP	.103
**3**	VDS	.063	VDS	.118	Mother job	.089	VDS	.068	DBP	.076	DBP	.098
**4**	PWG	.033	Baby sex	.030	SBP	.082	Mother job	.067	Maternal age	.043	PWG	.067
**5**	Maternal age	.030	Parity	.025	Baby sex	.060	baby sex	.066	PWG	.041	Maternal age	.039
**6**	Baby sex	.028	PWG	.019	DBP	.050	DBP	.056	Baby sex	.041	baby sex	.033
**7**	Mother job	.027	Maternal age	.018	Parity	.031	SBP	.055	Parity	.039	Moder job	.023
**8**	Parity	.022	Mother job	.011	PWG	.023	Parity	.049	Mother job	.038	Parity	.022

*SBP* Means of systolic blood pressure during pregnancy, *DBP* Means of diastolic blood pressure, *PWG* Pregnancy weigh gain, *VDS* Vitamin D status during the pregnancy, *GDM* Gestational diabetes mellitus, *PROM* Premature rupture of membranes, *MDI* Mean Decrease Impurity

**Fig. 4:**
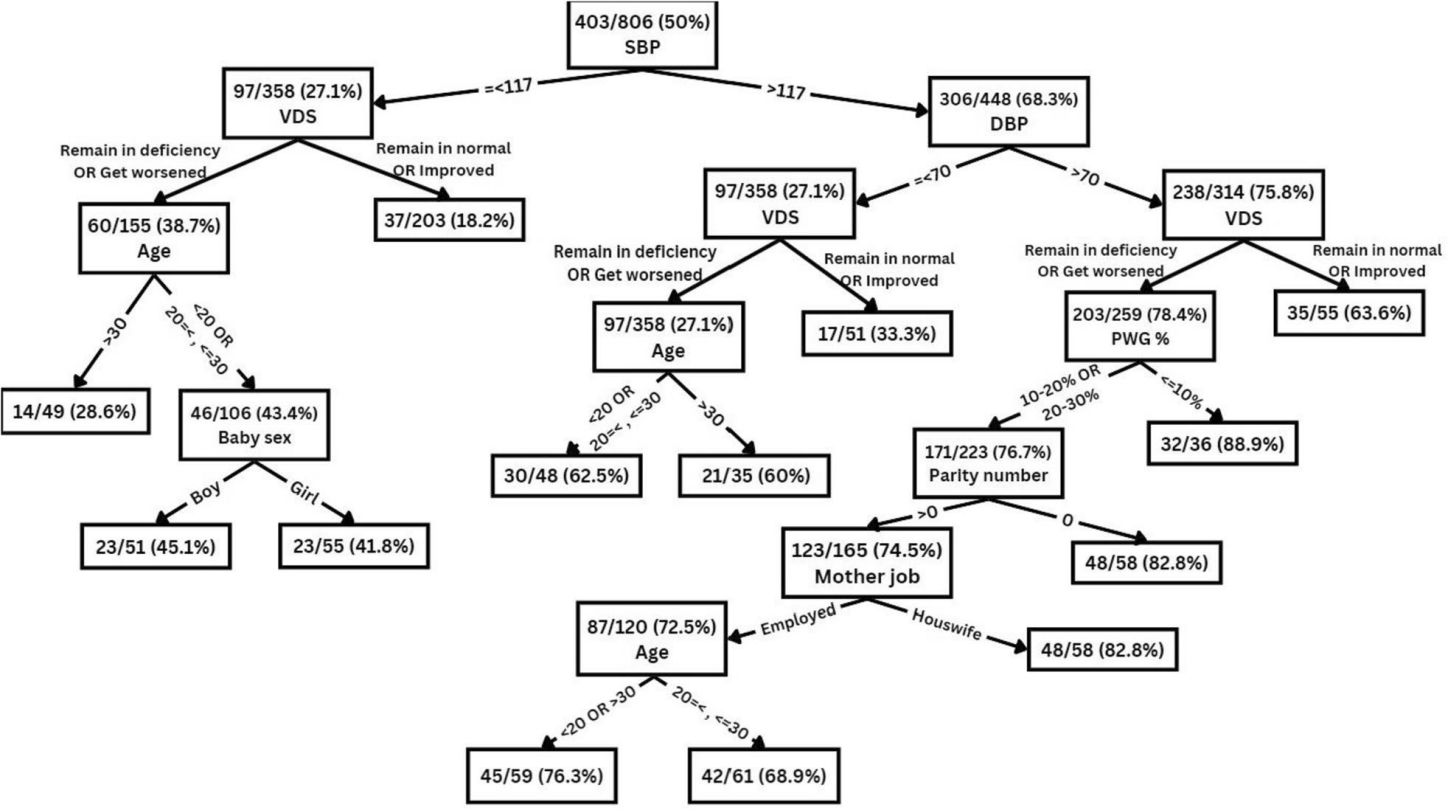
A decision tree from random forest for predicting maternal adverse events based on various predictors. SBP: Means of systolic blood pressure during pregnancy; DBP: Means of diastolic blood pressure; PWG: Pregnancy weigh gain; VDS: Vitamin D status during the pregnancy

Figure 4 shows one of the trees from the RF model and its prediction of MAE based on various features. This tree predicts the likelihood of a woman having a MAE based on her features. To use and interpret the tree, one can follow the path from the first node of the tree (named root node) to the leaf node that gives the final outcome or prediction for the observation. At each node, one can check the condition that splits the data and see which branch (left or right) is followed based on the feature value. The leaf node will provide the final outcome or prediction for the observation. The numbers in the boxes represent the number of samples with MAE, along with their corresponding percentage from that meet the criteria at each node. This aids in determining the MAE based on the features. These values provide insight into which features are most important for predicting MAE, and help to explain the decision-making procedure employed by the model.

The tree in Fig. 4 can be interpreted by starting from the root node and asking: Is the SBP less than or equal to 117? If yes, we move to the left child node and ask: Is the VDS normal or improved during pregnancy? If yes, we move to the right leaf node and predict that the woman has a low probability of having a MAE. If no, we move to the left leaf node and predict that the woman has a high probability of having a MAE. If the answer to the first question is no, we move to the right child node and continue with other features until we reach a leaf node that gives the final prediction.

## Discussion

In this study, we examined the influence of VDS during pregnancy, together with other predictors including maternal age, parity, sex of the baby, the mean SBP and DBP during pregnancy, and the PWG percentage, on MAE.

The results showed that higher rate of MAE was belong to women who remained deficient (35.2%) and those who worsened from sufficient to deficient (30.0%). The rates in women who improved (16.4%) or remained sufficient (11.8%) was in the next rank. This suggests that maintaining or improving vitamin D sufficiency during pregnancy may have protective effects against these complications. These findings are consistent with previous studies that have reported associations between low vitamin D levels and increased risks of adverse pregnancy outcomes [1, 20, 21]. However, the previous studies do not provide any specific results regarding the rates of MAE based on changes in VDS during pregnancy. The present study adds to the existing literature by showing that risk of adverse pregnancy outcomes in various status of changes in VDS over the course of pregnancy, rather than a single measurement, which may be more relevant for maternal health.

The predictors were ranked according to their impact in RF analysis, utilizing a metric referred to as variable importance, in the predicting of MAE (having one or more events) and each event individually. RF analysis revealed that SBP is the most important variable for MAE, followed by DBP. Hypertension is widely acknowledged as a prominent determinant for maternal adversities, including preeclampsia, eclampsia, placental abruption, and fetal growth restriction [22–24]. Following the blood pressure, the RF model indicated that alterations in VDS during pregnancy are among the most important factors. These findings suggest that the surveillance and management of vitamin D deficiency during pregnancy could potentially reduce the incidence of adverse outcomes. The insufficiency of Vitamin D has also been linked to increasing risks of gestational diabetes, preeclampsia, preterm birth, and cesarean section [25–27]. Although some studies have not fully confirmed the existence of these connections, for example the relationship between vitamin D deficiency and preeclampsia seems to be inconclusive [28]. However, many studies have been conducted in this field, but in general, due to the many contradictions that have been reported in the studies, it is challenging to reach a final decision. Considering that most of the current studies have measured vitamin D at one point and have not considered the changes of vitamin D during pregnancy, conducting well-designed interventional studies seems essential.

The variable importance scores also demonstrated that the role of VDS during pregnancy is a significant predictor for all outcomes. It was the third predictor for the overall MAE (having one or more events). It also emerged as the third predictor for preeclampsia, the second predictor for abortion, the third predictor for GDM, the second predictor for preterm delivery, and the first predictor for PROM. These findings highlight the importance of VDS during pregnancy for maternal and fetal health. There are various relevant articles which identified some risk factors that were associated with increased or decreased risk of MAE [29–33]. However, the authors of the study did not relative importance or contribution of these predictors to the final outcome. The presented study seems to be unique in that it ranks the predictors according to their influence within the models. This methodology enables to understand which predictors are more important than others, and how they interact with each other. This is a novel and valuable approach that offers valuable insights that can be utilized to enhance the quality of the findings. The investigation conducted by Muglia et al. maternal factors during pregnancy, such as obesity and smoking, can have adverse effects on maternal, fetal, and childhood outcomes [29]. Another investigation discovered that the outcomes for both the mother and newborn were related to heightened levels of blood pressure levels [33]. Additionally, the other investigation recognized various elements that contribute to the development of preeclampsia include familial lineage, advanced age, hypertension, diabetes, obesity, and other simultaneous ailments [34]. A review has also verified that mothers who are overweight face a higher risk for various complications [30]. Furthermore, another investigation has determined that diabetes type 1 and 2, chronic kidney disease and chronic hypertension were more strongly associated with preterm than term preeclampsia [32]. None of these have provided a quantitative assessment of importance or contribution of each predictor to the occurrence of MAE. Instead, they solely delineated the factors that increase the risk of MAE or compared the risk perception of different groups.

The utilization of novel techniques of examination, for instance, the RF methodology, opens a new window to access clinical data. The RF analysis provides a simple and intuitive way to identify the most influential variables for classifying the outcome. By using this approach, we were able to identify specific groups of women who are at higher risk of adverse events and develop targeted interventions to improve their outcomes. In the literature review, no article was found that used this method to predict MAE, so we could not compare our results with the current literature.

One of the limitations of the present study is that there may be some influencing variables that were not available in the data, such as maternal lifestyle, socio-economic status, smoking, underlying diseases, genetic and etc., that could affect the outcomes. Therefore, future studies with the presence of more variables are suggested to control for these potential confounders and to explore the causal mechanisms of VDS during pregnancy. Another limitation of the present study is that the VDS was measured using a single blood sample at baseline and delivery, which may not reflect the true VDS throughout the pregnancy. Vitamin D levels can vary depending on various factors such as seasonal changes and dietary intake. Therefore, future studies with repeated measurements of vitamin D at different stages of pregnancy are suggested to consider the dynamic changes of VDS and their effects on maternal health outcomes.

## Conclusion

This study highlights the importance of VDS during pregnancy for maternal and fetal health. It also indicates that vitamin D supplementation may be a potential preventive strategy for reducing the risk of MAE. However, further studies are needed to confirm the causal relationship between VDS and MAE, and to determine the optimal dose and timing of vitamin D supplementation.

## Data Availability

Derived data supporting the findings of this study are available from the corresponding author.

## Abbreviations

VDS: Vitamin D status
MAE: Maternal adverse events
SBP: Systolic blood pressure
DBP: Diastolic blood pressure
PWG: Pregnancy weight gain
GDM: Gestational diabetes mellitus
PROM: Premature rupture of membranes
RF: Random forest
